# A rapid and sensitive bioassay to measure bone morphogenetic protein activity

**DOI:** 10.1186/1471-2121-8-41

**Published:** 2007-09-19

**Authors:** Lior Zilberberg, Peter ten Dijke, Lynn Y Sakai, Daniel B Rifkin

**Affiliations:** 1 Department of Cell Biology, New York University School of Medicine, New York, New York 10016, USA; 2 Department of Molecular and Cell Biology, Leiden University Medical Center, 2300 RC Leiden, The Netherlands; 3 Department of Biochemistry and Molecular Biology, Oregon Health & Science University and Shriners Hospital for Children, Portland, OR 97239, USA; 4 Department of Medicine, New York University School of Medicine, New York, New York 10016, USA

## Abstract

**Background:**

Bone morphogenetic proteins (BMPs) are members of the TGF-beta superfamily and were originally identified as proteins that induce ectopic bone formation. BMPs were shown subsequently to be involved in several biological processes during development and in adult tissues through the regulation of the growth, differentiation and apoptosis of various cell types. An alkaline phosphatase (ALP)-based assay is the most widely used assay to evaluate BMP activity. However, the ALP assay is not rapid and not sensitive enough to measure BMP activity at physiological concentrations. In this paper, we describe a highly sensitive, rapid, and specific cell-based assay for the quantification of BMP activity.

**Results:**

Two cells lines, C2C12 and HepG2 were stably transfected with a reporter plasmid consisting of BMP-responsive elements from the Id1 promoter fused to a luciferase reporter gene. Exposure of cells containing this construct to BMPs induces the expression of luciferase, which can be quantified with a luminometer. The bioassay is specific for BMPs and can detect BMP-4 activity at a concentration as low as 3 pM. Related family members, such as TGF-beta1, TGF-beta2 and TGF-beta3, do not induce the reporter gene.

**Conclusion:**

The assay is rapid (less than 24 hours) and can be used, as described in this paper, to measure BMP activity in complex solutions and in cell culture in a simple and efficient way.

## Background

Bone morphogenic proteins (BMPs) are members of the transforming growth factor beta (TGF-β) superfamily that consists of a number of structurally related polypeptides that control a broad array of cellular processes, including cell proliferation, apoptosis and differentiation [1,2]. Although BMPs originally were identified as proteins that induced formation of bone when implanted into the muscle of adult rats [3,4], BMPs also play crucial roles in dorsoventral patterning of the mesoderm, neural patterning, skeletal development, and limb formation [5]. Altered BMP signaling pathways are associated with several human diseases including arthritis, osteoporosis, kidney diseases, cancer and pulmonary hypertension [6-10].

BMPs are synthesized as precursor proteins. After dimerization, the precursor molecules are proteolytically cleaved within the cell by proprotein convertases at the multibasic motif RXXR to yield the active, carboxy-terminal mature protein dimer [11-13]. BMPs exert their biological activity through combinations of type I and type II serine/threonine kinase receptors. Three distinct BMP type I receptors, i.e. activin receptor-like kinase (ALK)-2, ALK3 (also termed BMPRIA) and ALK6 (BMPRIB) and three distinct type II receptors, i.e. BMP type II receptor (BMPRII) and two activin type II receptors (ActR-IIA and ActR-IIB) have been identified [1]. Upon ligand binding, the type II receptors phosphorylate the type I receptor, which in turn initiates the downstream signaling process through phosphorylation of BMP-specific receptor-regulated Smads (R-Smads), i.e. Smad1, Smad5 or Smad8 [14-16]. R-Smads form a complex with Smad4 and translocate into the nucleus where the complex regulates the transcription of various target genes through its association with other transcription factors [17]. In contrast, TGF-β and activin signaling pathways recruit Smad2 and Smad3, which can also form heteromeric complexes with Smad4.

In addition to the tissue-specific expression of BMPs and their surface receptors, the biological activity of BMPs is regulated by a number of extracellular inhibitory molecules, such as chordin, noggin and follistatin [18]. Moreover, BMP signaling can be modulated by cross-talk with other pathways. TGF-β and BMP can modulate the activity of each other through competition for the interaction of their respective R-Smad with Smad4 or through activation of inhibitor Smads (I-Smads) [19,20].

The biological effects of BMPs have been used to develop quantitative bioassays. To date, alkaline phosphatase activity, a marker of osteoblast differentiation, measured in mesenchymal cells upon BMP stimulation, is the in-vitro assay most widely used among the research community to evaluate BMP activity [21]. However, the response in this assay is slow, usually taking between two to five days, and the assay is not sensitive enough to measure the low BMP concentrations generated in many biological systems.

Here we describe the development of a rapid and highly sensitive cell-based assay to measure BMP activities in different biological contexts. C2C12 mouse mesenchymal cells and HepG2 human hepatoma cells were stably transfected with a construct consisting of a BMP/Smad-dependent specific enhancer derived from the Id1 promoter and fused to a luciferase reporter gene [22]. Through screening and cloning, we generated two stable cell lines, C2C12BRA and HepG2BRA, which can measure BMP activity with high sensitivity and in various biological situations.

## Results

### Expression of BMP and BMPRs in the HepG2BRA and C2C12BRA

C2C12 mouse myoblast and HepG2 human hepatoma cell lines, which are known to respond to BMPs, were stably transfected with the BRE-Luc construct. This construct contains regions of the mouse Id1 promoter, which are important for the induction of Id1 transcription by BMPs, fused to a luciferase reporter gene. After cloning the transfected C2C12 and HepG2 colonies, cells from C2C12 clone 9 (named C2C12BRA) and HepG2 clone 15 (named HepG2BRA) were found to be the most sensitive upon stimulation with recombinant BMP-4 (data not shown). These clones were used for all subsequent experiments.

**Figure 1:**
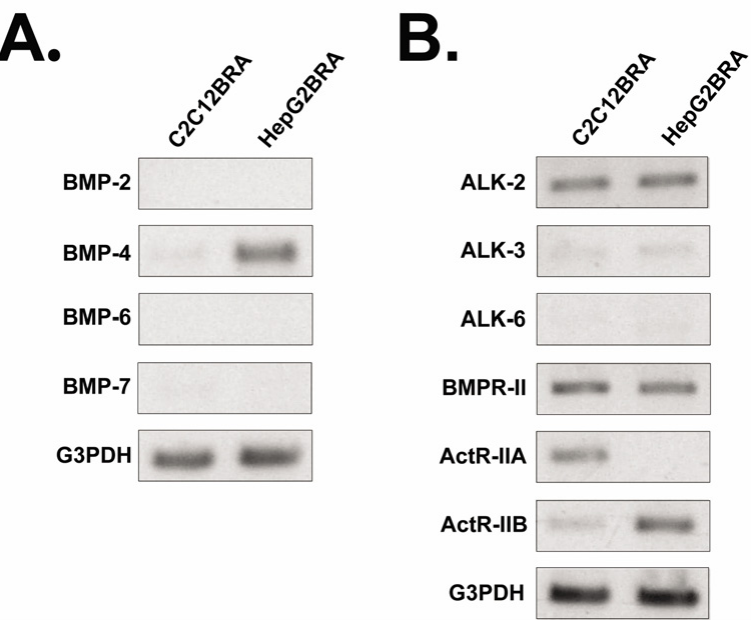
**Expression of BMP and BMPRs in HepG2BRA and C2C12BRA cells**. Total RNA from C2C12BRA and HepG2BRA cells was isolated and used as a template for the reverse transcriptase-polymerase chain reaction (RT-PCR) to examine the expression of BMP and BMPRs. G3PDH gene expression was used as an internal control. PCR products were separated in 1% agarose gel and visualized after ethidium bromide staining. (A) Representative RT-PCR analysis of total RNA using specific primers for BMP-2, BMP-4, BMP-6 and BMP-7. (B) Representative RT-PCR analysis of total RNA using specific primers for BMP type I receptors (ALK2, ALK3, ALK6) and BMP type II receptors (BMPR-II, ActR-IIA, ActR-IIB). Similar results were obtained in two separate experiments.

To obtain a comprehensive picture of the repertoire of BMPs and BMPRs expressed by C2C12BRA and HepG2BRA cells, we analyzed the expression of mRNAs coding for BMPs and BMPRs. Total RNA was isolated from C2C12BRA and HepG2BRA cells and subjected to RT-PCR. HepG2BRA cells expressed the mRNA for BMP-4 (Fig. 1A). No PCR products were observed for BMP-2, BMP-6 and BMP-7. We next analyzed the expression of mRNA coding for the three type I receptors (ALK2, ALK3 and ALK6) and the three type II receptors (ActR-IIA, ActR-IIB and BMPRII). C2C12BRA cells express ALK2, BMPRII and ActR-IIA mRNA (Fig. 1B). Trace levels of ALK3 and ActR-IIB were detected, whereas no expression of ALK6 was observed. HepG2BRA cells express ALK2, BMPRII and ActR-IIB mRNA. Trace levels of ALK3 were detected, whereas no PCR products were obtained for ALK6 and ActR-IIA (Fig. 1B).

### Dose-dependent induction of the BRE-Luc contruct by BMPs

**Figure 2:**
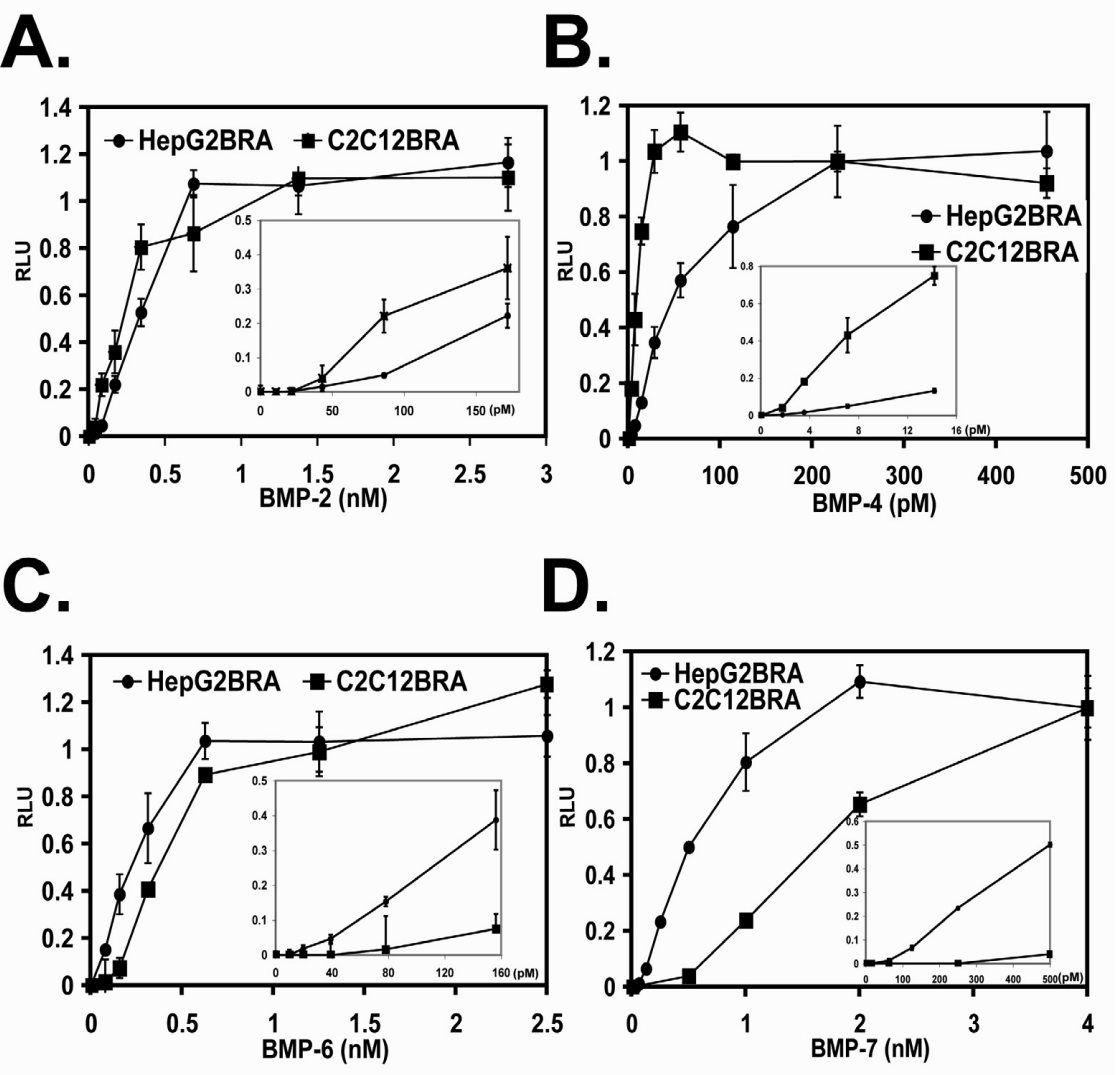
**Dose-dependent induction of the BRE-Luc construct by BMPs**. C2C12BRA and HepG2BRA cells were added to 96-well plates at 4 × 10^3 ^cells per/well and 5 × 10^3 ^cells/well, respectively. The reporter cells were incubated with increasing concentrations of the indicated recombinant BMPs in DMEM, 0.1% BSA. After 15 h incubation, BMP activity was assessed by measuring luciferase activity in the cell lysates. (A) BMP-2. (B) BMP-4. (C) BMP-6. (D) BMP-7. Background luciferase level in the control sample (no BMP) was subtracted from each of the experimental values. The results are expressed as relative luciferase units (RLU). Each point represents the mean ± SEM of triplicate wells from 1 representative experiment. Similar results were obtained in two separate experiments.

HepG2BRA and C2C12BRA cells were assayed for the induction of luciferase expression using a broad range of BMP-2, BMP-4, BMP-6 and BMP-7 concentrations (Fig. 2). HepG2BRA and C2C12BRA cells demonstrated equivalent dose-responses when stimulated with BMP-2 (ED_50 _~ 0.25–0.35 nM; sensitivity 40–80 pM) (Fig. 2A) and BMP-6 (ED_50 _~ 0.2–0.4 nM; sensitivity 40–150 pM), but a different dose-response curve for BMP-4 and BMP-7 (Fig. 2B, 2C and 2D, respectively). BMP-4 was the most potent inducer of luciferase activity with an ED_50 _of approximately 10 pM and 50 pM for C2C12BRA and HepG2BRA cells, respectively. BMP-7 was the least potent inducer of luciferase expression with an ED_50 _of approximately 0.5 nM and a sensitivity between 0.1 to 0.15 nM for the HepG2BRA reporter assay. The dependence of the assay for the ligand was shown for BMP-4 by inclusion of a neutralizing antibody for BMP-4 (MAB 757) that blocked the luciferase response (data not shown).

### Effect of incubation time on luciferase activity

**Figure 3:**
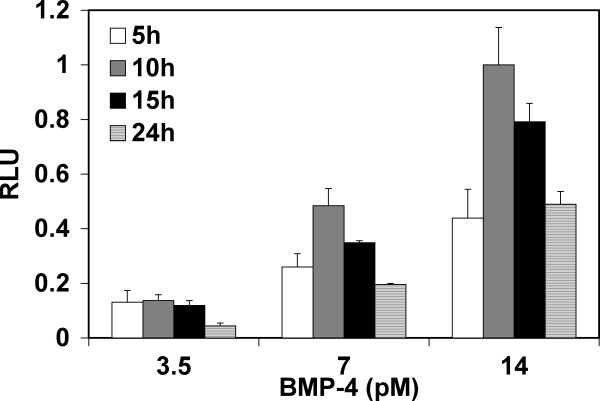
**Effect of incubation time on BMP assay sensitivity**. C2C12BRA cells were plated at 4 × 10^3 ^cells/well. Recombinant BMP-4 at the indicated concentrations was incubated with the cells for 5, 10, 15 or 24 h prior to assaying for luciferase activity. Background luciferase level in the control sample (no BMP) was subtracted from each of the experimental values. The results are expressed as relative luciferase units (RLU) (the activity with 14 pM of BMP-4 after 10 h incubation time equals one). Each point represents the mean ± SEM of triplicate wells from one representative experiment. Similar results were obtained in two separate experiments.

Next we tested the response of C2C12BRA cells to recombinant BMP-4 using different incubation times and different concentrations of the growth factor. At a concentration of recombinant BMP-4 as low as 3.5 pM, the sensitivity of the C2C12BRA cells was equivalent for incubation times of 5 h, 10 h and 15 h but decreased at an incubation time of 24 h (Fig. 3). When C2C12BRA cells were stimulated with concentrations above 3.5 pM, incubation times between 10 h and 15 h were optimal. Shorter or longer incubation times yielded decreased responses (Fig. 3). It is interesting that the luciferase response declined at the longer time points. The reasons for this are unclear but may relate to the kinetics of gene expression and stability of the luciferase.

### Specificity of the assay

**Figure 4:**
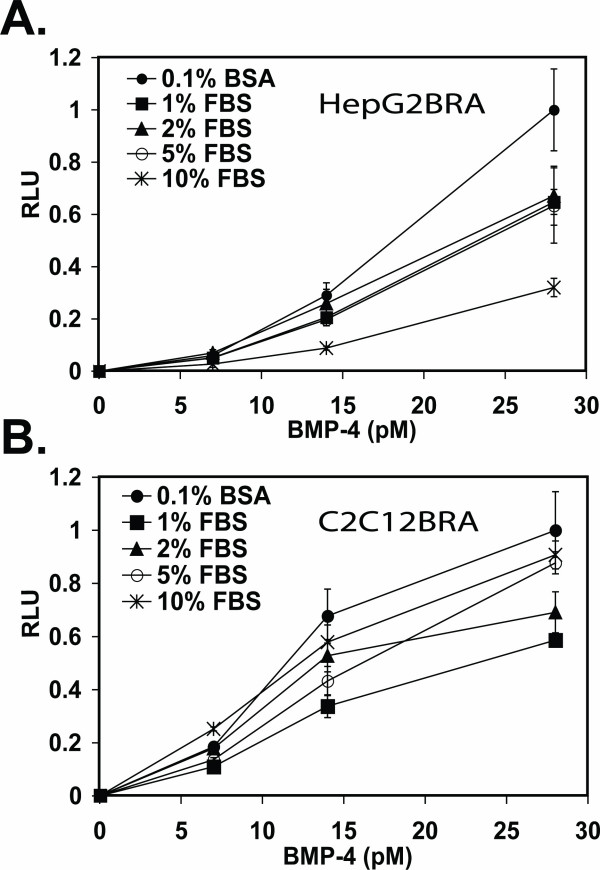
**Effect of serum on the assay**. HepG2BRA (A) and C2C12BRA (B) cells were incubated with DMEM containing the indicated concentration of rBMP-4 with 0.1% BSA or 1%, 2%, 5% and 10% FBS. Luciferase activity was measured after 15 h of incubation. Background luciferase level in the control sample (no BMP) was subtracted from each of the experimental values. The results are expressed as relative luciferase units (RLU). Each point represents the mean ± SEM of triplicate wells from one representative experiment. Similar results were obtained in two separate experiments.

Next we investigated the effect of different concentrations of serum on the stimulation of luciferase activity with recombinant BMP-4. The responses of HepG2BRA and C2C12BRA cells differed with respect to serum concentrations. The presence of serum impaired the response of HepG2BRA cells at all concentrations of rBMP-4 tested, with a strong decrease in sensitivity with medium containing 10% FBS and a mild decrease with serum concentrations of 1%, 2% and 5% (Fig. 4A). With HepG2BRA cells, the luciferase response was best using serum-free medium and BSA added at a final concentration of 0.1% (DMEM-BSA). On the other hand, 10% serum had only a minor effect on the response of C2C12BRA cells compared to incubation with DMEM-BSA medium (Fig. 4B). At a low BMP-4 concentration (7 pM), the relative luciferase activity in the presence of 10% FBS was actually higher compared to that with serum-free medium. Addition of 1% or 2% serum shifted the curve downward at all BMP-4 concentrations tested (Fig. 4B).

**Figure 5:**
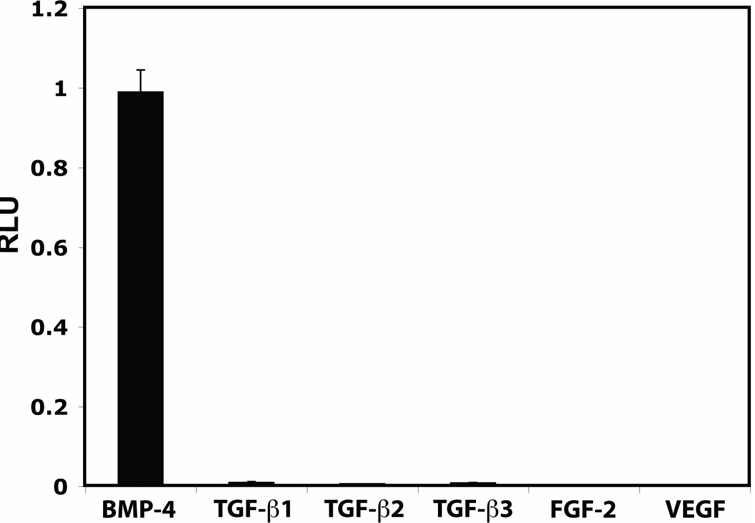
**Effects of growth factors on the reporter-cell assay**. C2C12BRA cells were stimulated with 1ng/ml (TGF-β1, TGF-β2 and TGF-β3) or 10 ng/ml (FGF-2 and VEGF) of growth factors and the luciferase activity measured after 15 h. Treatment with 14 pM of rBMP-4 served as a positive control. Background luciferase level in the control sample (no BMP) was subtracted from each of the experimental values. The results are expressed as relative luciferase units (RLU). Each point represents the mean ± SEM of triplicate wells from one representative experiment. Similar results were obtained in two separate experiments.

We also tested the induction of luciferase activity with other members of the TGF-β superfamily, such as TGF-β1, TGF-β2, TGF-β3, as well as TGF-β-unrelated growth factors, such as FGF-2 and VEGF. As shown in Fig. 5, although BMP-4 at 14 pM induced a strong luciferase expression, there was no elevation of luciferase activity when cells were incubated with different isoforms of TGF-β even at concentration as high as 1 ng/ml. 10 ng/ml of bFGF or VEGF did not induce luciferase expression.

**Figure 6:**
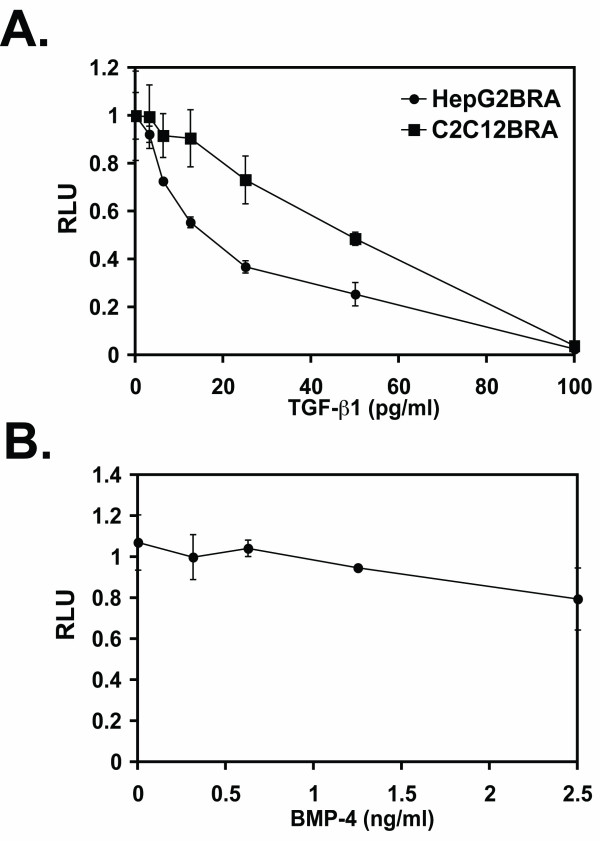
**Effect of TGF-β1 on BMP-4 signaling pathway**. (A) C2C12BRA and HepG2BRA cells were treated with 14 pM of rBMP-4 in combination with increasing concentrations of TGF-β1. After 15 h, BMP activity was assessed by measuring luciferase activity in the cell lysates. Background luciferase level in the control sample (no BMP) was subtracted from each of the experimental values. The results are expressed as relative luciferase units (RLU) (the activity with 14 pM BMP-4 alone equal to one). (B) TGF-β-responsive reporter cells (TMLC) were stimulated with 250 pg/ml of TGF-β1 and increasing concentrations of BMP-4. After 16 h, TGF-β1 activity was assessed by measuring luciferase activity in the cell lysates. Each point represents the mean ± SEM of triplicate wells from one representative experiment. Similar results were obtained in two separate experiments.

It is well established that TGF-β and BMP can modulate the activity of each other through cross-talk of their respective pathways [20]. Therefore, we examined whether TGF-β1 would affect the induction of luciferase activity by recombinant BMP-4. As shown in Fig. 6A, increasing the concentration of active TGF-β1 reduces the luciferase activity induced by BMP-4 with both C2C12BRA and HepG2BRA cells. Similar results were obtained with TGF-β2 and TGF-β3 (Data not shown). We performed the opposite experiment using TGF-β-responsive reporter cells (TMLC) that produce luciferase activity in response to TGF-β [23]. TMLC were co-stimulated with 250 pg/ml of TGF-β1 and increasing concentrations of BMP-4 (Fig. 6B). Compared to the effect of TGF-β on the BMP-responsive reporter cells, BMP-4 had a very slight effect on luciferase activity induced by TGF-β1 in TMLC cells even at a concentration as high as 2.5 ng/ml.

### Measurement of BMP activity from biological samples

**Figure 7:**
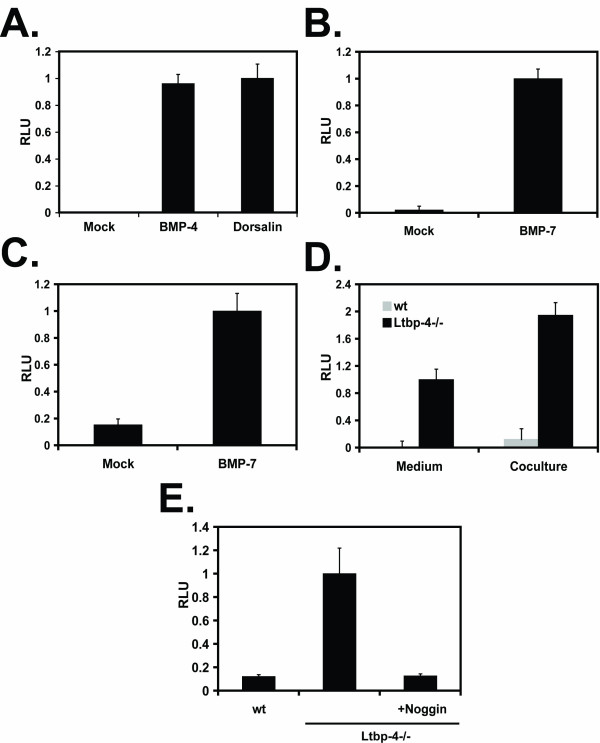
**BMP activity measured in biological samples**. (A) 4 × 10^3 ^C2C12BRA cells/well were treated with supernatants of COS cells transfected with either a BMP-4 or a Dsl expression vector. Empty pcDNA3 plasmid was used as a mock control (B) 4 × 10^3 ^C2C12BRA cells/well were treated with supernatants of 293T cells transfected with the empty plasmid pcDNA3 (mock) or BMP-7 expression vector. (C) 4 × 10^3 ^C2C12BRA cells/well were cocultured with 4 × 10^3 ^293 cells/well stably transfected with a pcDNA3 (mock) or a BMP-7 expression plasmid. After 24 h, luciferase activity was measured. (D) 4 × 10^3 ^C2C12BRA cells/well were cocultured with 1 × 10^4 ^wt or *Ltbp-4 *hypomorphic (*Ltbp-4*^-/-^) lung fibroblasts. Luciferase activity was measured after 24 h. Conditioned medium from the coculture was collected from wt and *Ltbp-4*^-/- ^lung fibroblasts and applied to C2C12BRA cells for 15 h to measure the amount of BMP activity. (E) 5 × 10^3 ^wt or *Ltbp-4 *hypomorphic (*Ltbp-4*^-/-^) lung fibroblasts were added to a 96 well plate. After 24 h, 4 × 10^3 ^C2C12BRA cells were added with or without 2 μg/ml of recombinant noggin. Luciferase activity was measured after 24 h. The results are expressed as relative luciferase units (RLU). Each point represents the mean ± SEM of triplicate wells from one representative experiment. Similar results were obtained in two separate experiments.

We tested the BMP assay with conditioned medium from COS cells transiently transfected with either a dorsalin-1 (Ds1-1) or a BMP-4 expression plasmid. Dorsalin is member of the BMP family that regulates dorsoventral patterning of the neural tube. Like BMPs, Dsl-1 induces alkaline phosphatase synthesis in an osteoblast differentiation assay [24]. We found that both Dsl-1- and BMP-4-conditioned medium induced the expression of luciferase, whereas mock conditioned medium had no effect (Fig. 7A). Conditioned medium from 293T cells transfected with a BMP-7 expression plasmid led to a substantial increase in luciferase activity compared to mock conditioned medium (Fig. 7B). We also cocultured C2C12BRA cells with 293 cells stably transfected with BMP-7 expression plasmid and observed a substantial induction of luciferase activity compared to 293 cells stably transfected with an empty plasmid (Fig. 7C).

We determined if the reporter assay could be used to detect and quantify BMP activity in biological samples without any enrichment, i.e. concentrating the conditioned medium or overexpressing BMP in cells. For this purpose we measured BMP activity in conditioned medium from lung fibroblasts established from wt and *Ltbp-4 *hypomorphic mice (wt and *Ltbp-4*^-/- ^cells, respectively). It was reported that *Ltbp-4*^-/- ^lung fibroblasts express more BMP-4 and less gremlin, a BMP inhibitor, compared to the wt cells [25]. As shown in Fig. 7D, conditioned medium from *Ltbp-4*^-/- ^cells induced luciferase activity more efficiently than wt cells in C2C12BRA cells. The difference between wt and *Ltbp-4*^-/- ^cells corresponded to a BMP-4 concentration of ~3.5 pM. We also cocultured C2C12BRA cells with wt and *Ltbp-4*^-/- ^lung fibroblasts and again observed more BMP activity in *Ltbp-4*^-/- ^cells compared to wt cells (Fig. 7D). In fact, the coculture assay yielded almost twice the activity as the assay using conditioned medium. Inclusion in the assay of noggin, an inhibitor of BMP, blocked the BMP activity observed in *Ltbp-4*^-/- ^cells (Fig. 7E).

## Discussion

Currently, the ALP assay is the most widely and routinely used cell-based assay to measure BMP activity. ALP expression is a marker of osteoblast differentiation and BMP-induced osteogenic differentiation of mesenchymal cells. However, this assay has several drawbacks. The time required to perform the assay is long, usually between two to five days. This assay is not sensitive enough to detect BMP concentrations generated in many biological systems and ALP expression can be affected negatively or positively by a variety of other signaling pathways, such as Sonic hedgehog, FGF-2 or Wnt/beta-catenin [26-28].

In the present paper, we describe the establishment of a highly sensitive and specific assay to measure BMP activity. Two stable cell clones, C2C12BRA and HepG2BRA, were isolated after transfection of C2C12 and HepG2 cells with an expression construct containing distinct sequence motifs derived from the mouse Id1 promoter fused to a luciferase gene. When compared to other available cell-based bioassays or protein-based assays, our assay provides improved sensitivity for several BMP types, is faster, and is more specific for the measurement of BMP activity in complex biological solutions and in various biological situations.

Recently, another cell-based assay was described using C3H10T1/2 embryonic mouse cells stably transfected with the same reporter construct that we used in our assay [29]. Although the C3H10T1/2 assay can detect recombinant BMP-2 in DMEM-BSA at twofold lower concentration than our assay, our assay is more sensitive when measuring the activity of BMP-4 (3 pM compared to a concentration >250 pM) and BMP-7 (125 pM compared to a concentration >300 pM). Moreover, the sensitivity of the C3H10T1/2 assay was diminished when performed in the presence of large excess of foreign protein, i.e. concentrations of serum above 2%. Although the sensitivity of HepG2BRA cells was also decreased by increasing concentration of serum, the ability of C2C12BRA cells to measure concentrations of recombinant BMP-4 as low as 7 pM was not affected by concentrations of serum as high as 10%. Thus, depending upon the biological medium to be assayed, one could use either C2C12BRA or HepG2BRA cells. Finally, we show how the assay sensitivity can be enhanced by cocultures of test cells and reporter cells.

Radioimmunoassays and enzyme linked immunoabsorbent assays (ELISA) are sensitive and specific and have been developed for BMPs [30,31]. The disadvantages of these assay are that they are expensive and that they measure both biologically active and inactive BMPs. Recently, a new protein based assay named ELIRA (enzyme-linked immunoreceptor assays) was developed for quantification of recombinant BMP-2 [32]. Although this assay measures the quantity of BMP-2 that binds to its receptors, this binding does not necessarily mean that the protein is biologically active. Our assay quantifies only BMP that is biologically active as opposed to immuno-reactive BMP. Moreover, the lower detection limit of the ELIRA was high (~11 nM) compared to our assay and assay performance was disturbed by the presence of foreign proteins [32].

The differential sensitivity of C2C12BRA and HepG2BRA cells observed for different BMPs might be explained by the different expression patterns of endogenous BMPs and BMP receptors as characterized by RT-PCR (Fig. 1). Distinct BMPs have different effects depending on the nature, the absolute number and the different combinations of BMP type I and type II receptors on the cell surface [1]. The higher sensitivity of C2C12BRA cells compared to HepG2BRA cells when measuring BMP-4 activity might be due to the lower endogenous expression of BMP-4 in C2C12BRA cells compared to HepG2BRA cells (Fig. 1A). Although our assay is not BMP-isoform specific, the appropriate addition of neutralizing antibodies for a given BMP and the use of a standard curve should allow the quantification of specific biologically active BMPs.

As shown in Fig. 6A, whereas our assay is specific for BMP, luciferase expression induced by BMP can be affected by active TGF-β. To our knowledge, this is the first time that this observation has been made for this BMP reporter construct. Although TGF-β does not have an effect on Id expression by itself, TGF-β might indirectly modify the effect of BMP by sequestering Smad4 or induction of the expression of I-Smads [19,20,22]. TGF-β activity measured with a TGF-β reporter assay was not affected by recombinant BMP-4 (Fig. 6B). This is likely due to the lack of functional BMP-Smad pathway in these cells. Interestingly, the sensitivity of C2C12BRA for BMP-4 is only minimally affected by high concentrations of serum known to contain TGF-β (Fig. 4B). This observation can be explained by the fact that TGF-β in serum is in a latent form that must be activated in order to bind to its receptor and thus interfere with the BMP signaling pathway [33].

Beside its high sensitivity and specificity, another major advantage of our assay is the possibility to measure BMP activity from biological samples in a simple and efficient way. Quantification of BMP activity in complex biological solutions, as opposed to a defined medium, such as DMEM-BSA, is necessary for a cell-based assay to be useful to address specific problems related to BMP activity. In this paper, we describe the use of this assay in several ways to enhance its utility. First, we tested the possibility of this assay to measure BMP activity in non-defined medium. Our BMP reporter assay detected BMP activity in the conditioned medium of COS and 293T cells transfected with BMP-4 and BMP-7 expression plasmids, respectively (Fig. 7A and 7B respectively). Interestingly, the amount of BMP activity detected in the conditioned medium was highly dependent on the cell line used to overexpress the specific BMP isoform. This might be due, as mentioned in previous studies, to the cell-specific expression pattern of proprotein convertases involved in the processing of the BMP inactive precursor form [34]. Second, C2C12BRA cells measure BMP activity when cocultured with 293 cells stably transfected with a BMP-7 expression vector (Fig. 7C). The advantage of this arrangement is that one can measure very low concentrations of BMP because of an increase in the local concentration of the cytokine without much dilution in the media. Third, this assay can measure endogenous BMP secreted by cells. Disruption of the *Ltbp-4 *gene in mice results in abnormal lung development due to an enhanced activation of the BMP-4 signaling pathway both in vitro and in vivo [25]. Microarray analysis revealed that isolated *Ltbp-4*^-/- ^lung fibroblasts from these mice express more BMP-4 and less of its inhibitor gremlin compared to wt cells [25]. Here we confirm these findings by showing that *Ltbp-4*^-/- ^cells produce more BMP activity than wt cells (Fig. 7D). Interestingly, coculture of the BMP reporter assay cells with *Ltbp-4*^-/- ^cells detected more BMP activity than when reporter cells were incubated with conditioned medium. This is probably due to an increase in the local concentration of BMP-4 without dilution of the cytokine in the media. The difference in luciferase activity between wt and *Ltbp-4*^-/- ^cells was BMP-specific as the inclusion of noggin, a specific BMP inhibitor, in the coculture blocked the luciferase response induced by *Ltbp-4*^-/- ^cells (Fig. 7E).

## Conclusion

We have generated a rapid, specific and sensitive cell-based bioassay that can be used to measure BMP activity in complex solutions and in various biological contexts. Moreover, this bioassay can be used to study BMP signaling and to screen and identify new targets to modify BMP signaling pathways either positively or negatively.

## Methods

### Cell lines and reagents

C2C12, HepG2 (obtained from Dr. A. Kumar; New York University, NY), COS, 293, and 293T cells were grown in Dulbecco's Modified Eagle's Medium (DMEM) and supplemented with 10% fetal calf serum and antibiotics. Mink lung epithelial cells (TMLC) stably transfected with a plasmid containing the luciferase cDNA downstream of a TGF-β-sensitive portion of the plasminogen activator inhibitor 1 promoter were used as described [23]. Lung fibroblast cultures from adult wt and *Ltbp-4*^-/- ^mice were obtained from Dr. K. Koli (University of Helsinki, Helsinki, Finland) and used as described [25]. 293 cells stably transfected with a BMP-7 expression vector have been previously described [35].

Recombinant human BMP-2, -4, -6, -7 and mouse noggin were purchased from R&D Systems Inc. Recombinant FGF-2 and VEGF were a gift from Dr. P. Mignatti (New York University School of Medicine, New York, NY 10016, USA).

pcDNA3 vector was obtained from Invitrogen (Carlsbad, CA, USA). The pMT21 expression vector containing Myc-tagged chick Dorsalin [24] was a gift from Dr. T. Jessell (Columbia University, NY). BMP-4 expression vector was a gift from Dr. A.H. Brivanlou (The Rockefeller University, NY). pNeo-(BRE)_2_-Luc was generated by inserting a neomycin resistance gene into pGL3 (BRE)_2_-Luc vector [22].

### RNA extraction, reverse transcription and polymerase chain reaction (PCR)

Total RNA was isolated from cells with Trizol (Invitrogen). cDNA was generated from RNA with reverse transcriptase and used as a template for amplification with Taq polymerase. Each PCR reaction consisted of initial denaturation at 94°C for 30s. The cycling parameters were as follows: 94°C for 30 s, 59°C for 45 s and 72°C for 1 min. After 25 cycles, reaction underwent a final extension at 72°C for 5 min. Amplification products were electrophoresed on 1% agarose gels and visualized by ethidium bromide staining The primer sequences used for PCR amplification, based on published sequences, were as follows: 5'-TTTGGACCTGGCTAATGGAG-3' and 5'-GGCCACTTATTGTTGGCACT-3' for human ActR-IIa; 5'-CTCCCTCACGGATTACCTCA-3' and 5'-AGGGCAGCATGTACTCATCC-3' for Human ActR-IIb; 5'-AATTGGCTTCTCGTTGCACT-3' and 5'-CATCTCCCAGATCCATGCTT-3' for mouse ActR-IIa; 5'-CATCATCACGTGGAACGAAC-3' and 5'-CTTGTGGACAACCACCTCCT-3' for mouse Act-RIIb; 5'-CCTTGGATGAGCGTCCAGTTG-3' and 5'-AGCCGAGCCTCTGCATC-3' for BMPR-II; 5'-AGACACTCCAGTACCCAGCTG-3' and 5'-GCTGTGAGTCTTGCGGATGG-3' for ALK-2; 5'-TGGTTCAGCGAACTATTGCCA-3' and 5'-TCAGCCATGATGTAGGGCTGG-3' for ALK-3; 5'-AGTGGATCAGGCCTCCCTCGC-3' and 5'-CACTTCTGGAGGCATATAGCG-3' for ALK-6; 5'-ACTTCCAGAGATGAGTGGGA-3' and 5'-ATCTTGGTGCAAAGACCTGCT-3' for mouse BMP-2; 5'-ACTACCAGAAACGAGTGGGA-3' and 5'-ATCTTGGTGCAAAGACCTGCT-3' for human BMP-2; 5'-CAGATGTTTGGGCTGCGCC-3' and 5'-AGGTGAGTCACCTCAATGGC-3' for BMP-4; 5'-TTCCTCAACGACGCGGACAT-3' and 5'-CCACCATGAAGGGCTGCTT-3' for BMP-6; 5'-TGGACCTGTACAACGCCATG-3' and 5'-TGGTTGCTGGTGGCTGTGAT-3' for BMP-7. 5'-ACCACAGTCCATGCCATCAC-3' and 5'-TCCACCACCCTGTTGCTGTA-3' for G3PDH.

### TGF-β bioassay

To measure TGF-β activity, TMLC cells in 96 wells were incubated with recombinant protein diluted in serum-free DMEM-0.1% BSA. Sixteen hours later, TGF-β was assessed by measuring luciferase activity in cell lysates as described previously [23]. All experiments were done in triplicate and repeated at least two times with similar results.

### Cell transfection

COS and 293T cells were transiently transfected in 6-well dishes with 1 μg of DNA per well using Lipofectamine Plus. After 16 h, the medium was changed to serum-free OptiMEM (GIBCO BRL) and conditioned for 24 h. Conditioned medium was precleared by centrifugation and tested in the BMP reporter assay.

To generate BMP reporter cells, C2C12 and HepG2 cells were stably transfected with pNeo-(BRE)_2_-Luc. Briefly, HepG2 and C2C12 cells were seeded at 8 × 10^4 ^cells/well in 35 mm dish for 24 h at which point they were transfected with 1 μg of pNeo-(BRE)_2_-Luc DNA using Lipofectamine. Twenty-four hours later, cells were reseeded at different densities and selected for antibiotic resistance using 700 μg/ml geneticin. The resistant colonies were isolated, expanded, and tested in reporter cell assays. Clone 9 for C2C12 (named C2C12BRA) and clone 15 for HepG2 (named HepG2BRA) were found to be the most sensitive upon stimulation with recombinant BMP-4.

### BMP bioassay and BMP co-culture bioassay

Unless indicated, the BMP assay was performed as follows: C2C12BRA and HepG2BRA cells were added to 96-well plates culture dishes at 4 × 10^3 ^cells/well and 5 × 10^3 ^cells/well, respectively. The cells were allowed to attach overnight. The medium was replaced with the indicated medium containing the recombinant protein to be tested for BMP activity. After 14 h, cells were washed twice with PBS and cell extracts were prepared with 45 μl of 1× cell lysis buffer (BD Pharmingen). 35 μl of the lysate was transferred to a 96-well microplate and assayed for luciferase activity using a LUMIstar Galaxy luminometer (BMG Labtechnologies). Luciferase activity was reported as relative luciferase units (RLU). The response of the cells to BMPs remained constant for at least 10 passages. When cells reached the 10^th ^passage, they were replaced with cells recovered from a frozen early passage stock.

For co-culture experiments, test cells were added at the densities indicated (see figure legends) in 96-well plates and allowed to attach overnight. 4 × 10^3 ^C2C12BRA cells/well were seeded on top and allowed to attach. Wells were washed once with PBS and incubated with 100 μl of DMEM supplemented with 10% FBS. After 24 h, luciferase activity in cell extracts was measured. All results are expressed as relative luciferase unit (RLU), which is the value of the raw luciferase activity minus the background, in order to eliminate variations in calculations of fold induction due to differences in the background (no BMP) values between experiments. All assays were performed in triplicate and repeated at least two times with similar results.

## Abbreviations

ActR, activin receptor; ALK, activin like receptor kinase; BMP, bone morphogenetic protein; FGF-2, basic fibroblast growth factor; VEGF, vascular endothelial growth factor; TGF-β, transforming growth factor-β; LTBP, latent TGF-β binding protein; Id, inhibitor of differentiation; rBMP, recombinant BMP; BMPR, BMP receptor; Bre-Luc, BMP responsive element-luciferase; RT-PCR, reverse transcriptase – polymerase chain reaction; DMEM, Dabecco's modified Eagle's medium; ALP, alkaline phosphatase; TMLC, mink lung epithelial cells with a PAI-luciferase vector; PAI-1, plasminogen activator inhibitor 1; G3PDH, glycocaldehyde 3 phosphate dehydrogenase.

## Competing interests

The author(s) declare that there are no competing interests.

## Authors' contributions

LZ carried out all the experiments, coordinated and designed experiments, analysed data and prepared the manuscript. PtD and LS provided expert advice, provided reagents, and participated in the revisions of the manuscript. DBR conceived of the study, participated in its design and edited the manuscript. All authors read and approved the final manuscript.
